# Clonal Flux and Spread of *Staphylococcus aureus* Isolated from Meat and Its Genetic Relatedness to *Staphylococcus aureus* Isolated from Patients in Saudi Arabia

**DOI:** 10.3390/microorganisms11122926

**Published:** 2023-12-06

**Authors:** Dalal M. Alkuraythi, Manal M. Alkhulaifi, Abdulwahab Z. Binjomah, Mohammed Alarwi, Hind M. Aldakhil, Mohammed I. Mujallad, Saleh Ali Alharbi, Mohammad Alshomrani, Saeed Mastour Alshahrani, Takashi Gojobori, Sulaiman M. Alajel

**Affiliations:** 1 Department of Botany and Microbiology, College of Science, King Saud University, Riyadh 11451, Saudi Arabiamanalk@ksu.edu.sa (M.M.A.); 2 Department of Biology, College of Science, University of Jeddah, Jeddah 23445, Saudi Arabia; 3 Microbiology Department, Riyadh Regional Laboratory, Ministry of Health, Riyadh 12746, Saudi Arabiasaaalharbi@moh.gov.sa (S.A.A.);; 4 College of Medicine, AL-Faisal University, Takhassusi Street, Riyadh 11533, Saudi Arabia; 5 Computational Bioscience Research Center, Biological and Environmental Sciences and Engineering, King Abdullah University of Science and Technology, Thuwal 23955, Saudi Arabia; 6 Food and Drug Authority, Jeddah 22235, Saudi Arabia; mimujallad@sfda.gov.sa; 7 Department of Public Health, College of Applied Medical Sciences, King Khalid University, Abha 62529, Saudi Arabia; 8 Reference Laboratory for Microbiology, Executive Department for Reference Laboratories, Research and Laboratories Sector, Food and Drug Authority, Riyadh 12843, Saudi Arabia

**Keywords:** MRSA genetic characteristics, *Staphylococcus aureus*, retail meat, One Health approach, clonal lineages, MLST, *spa* types

## Abstract

In this study, we investigated both meat-derived and methicillin-resistant *Staphylococcus aureus* (MRSA), exploring their genetic relatedness to patient-derived MRSA isolates in Saudi Arabia. We collected 250 meat samples and identified 53 *S. aureus* isolates, with 79% being methicillin-sensitive *Staphylococcus aureus* (MSSA) and 21% being MRSA. Moreover, we included 80 clinically confirmed patient-derived MRSA isolates. We identified the most common *S. aureus* clone in both patients and retail meat. In meat, ST6 and ST97 were the most common clones in 55% of the MRSA isolates, and ST1153 and ST672 were the most common in 21% and 17% of the MSSA isolates. In patients, ST5 and ST6 were the predominant clones in 46% of the *S. aureus* isolates. CC5/ST5-SCCmecVc-t311 and CC361/ST672-SCCmecV-t3841 were common MRSA clones in both meat and patients. CC97 and CC361 clones were the second most prevalent *S. aureus* clones in meat and were relatively common in patients. Furthermore, we sequenced and characterized novel *S. aureus* strains ST8109, ST8110, and ST8111. The genomic similarities between meat- and patient-derived *S. aureus* isolates suggest that retail meat might be a reservoir for *S.aureus* and MRSA transmission. Therefore, a structured One Health approach is recommended for *S. aureus* dissemination, genetic characterization, antibiotic resistance, and impact on human health.

## 1. Introduction

*Staphylococcus aureus* is a Gram-positive bacterium that is commonly found in the nostrils and skin of humans and animals [1]. However, it is opportunistic and can cause a range of infections, from mild skin infections to severe sepsis [1]. The introduction of semi-synthetic *β*-lactams in the 1960s led to the observation of methicillin resistance in *S. aureus*, complicating the treatment of *S. aureus* infections [2,3]. In Saudi Arabia, Gram-positive cocci were used for a Saudi national surveillance investigation, identifying 32% of *S. aureus* as methicillin-resistant *S. aureus* (MRSA) [4]. In addition, the prevalence of MRSA has risen to 38% among hospital infections [5]. Antimicrobial-resistant bacteria also impact animals, as well as the food chain sectors related to animals, due to antibiotic misuse [6]. Livestock-associated MRSA (LA-MRSA) is the main MRSA reservoir outside of hospitals, colonizing calves, cows, sheep, and poultry [7,8]. Several hospital-acquired MRSA (HA-MRSA) and community-associated MRSA (CA-MRSA) lineages are known to cause infections in humans, including CC1, CC5, CC8, CC22, and CC97, which have become successful LA-MRSA clones [9]. In Saudi Arabia, due to its clinical significance, researchers concentrate more on HA-MRSA than other types of MRSA [10]. Due to the persistent genetic markers that increase community-associated strain virulence and promote related transmission, CA-MRSA clones (e.g., CC5, CC22, and CC80) outnumber HA-MRSA in Saudi Arabian hospitals [5,11]. Several genomic typing techniques (e.g., multilocus sequence typing (MLST) and staphylococcal protein A (*spa*) typing) have been developed for *S. aureus* characterization to establish a genomic relatedness and track circulating *S. aureus* clones [12]. In this study, we focused on meat and meat products as well-known and significant *S. aureus* reservoirs associated with multiple outbreaks [13]. Following their isolation, identification, and whole-genome sequencing, we determined the genetic background of the *S. aureus* isolates by MLST and *spa* type. Epidemiology studies require clear results, comparable across different regions, monitoring changes and trends in clonal lineages among closely related strains [12]. Although several studies have been conducted in Saudi Arabia addressing the molecular characterization of MRSA [14,15,16,17], data about MRSA and *S. aureus* clonal flux, as well as information on the genomic relation between human, animal, and food *S. aureus* isolates in Saudi Arabia are limited. Therefore, genomic analysis of various clones of *S. aureus* isolates obtained from humans and meat is needed. Additionally, MRSA surveillance at the human-animal-food interfaces must be maintained.

## 2. Materials and Methods

### 2.1. Sample Collection

From February to June 2022, 84 non-duplicates clinically identified as *S. aureus* routine cultures were obtained from inpatients at the bacteriology department in the Riyadh Regional Laboratory and Blood Bank. Additionally, 250 retail meat samples, including camel, beef, chicken, fish, and lamb, were collected from various meat retailers throughout Riyadh, Saudi Arabia, from October 2021 to March 2022. All meat samples were transported in sealed plastic wrap or the original packaging to the Reference Laboratory of Microbiology at the Saudi Food and Drug Authority. Microbiological analysis was conducted on these samples within 24 h of sampling.

### 2.2. Ethical Approval

The ethical approval (approval number H-01-R-053) of the study was obtained from the Institutional Review Board committee of King Saud Medical City, Riyadh, Saudi Arabia and the Institutional Biosafety and Bioethics Committee of King Abdullah University of Science and Technology (approval number 22IBEC051).

### 2.3. Identification and Confirmation of S. aureus and MRSA Isolates

Clinical isolates were identified as MRSA using VITEK2 (bioMérieux, Craponne, France) and BD Phoenix (BD Diagnostics, Franklin Lakes, NJ, USA). Meat samples were processed by homogenizing them in a stomacher using a sterile plastic bag. This involved 25 g of each meat sample and 225 mL of buffered peptone water with 6.5% NaCl. The Petrifilm^TM^ Staph Express count plate (3M^TM^, St. Paul, MN, USA) and Petrifilm™ Staph Express disk were used to distinguish *S. aureus* colonies from other *Staphylococci*. Single *S. aureus* colonies were then transferred onto mannitol salt agar (Neogen, Lansing, MI, USA). *S. aureus* was identified and confirmed using the classical method, which includes Gram stain, catalase test, and coagulase test (PROLEX^TM^, Neston, UK). All *S. aureus* isolates were further identified by a matrix-assisted laser desorption/ionization time-of-flight mass-spectrometer (MALDI-TOF) (Bruker, Bremen, Germany) using the 70% formic acid protein extraction method. 

### 2.4. Culture-Based Method

MRSA chromogenic agar is used for the selective and differential detection of MRSA. Individual colonies of *S. aureus* isolates are transferred to harlequin MRSA chromogenic agar (Neogen, Lansing, MI, USA). This medium allows MRSA to grow because it includes a cefoxitin (Fox) supplement. The α-glucosidase enzyme produced by *S. aureus* cleaves the chromogenic substrate, resulting in blue-colored colonies.

### 2.5. Molecular-Based Methods

DNA was extracted from the bacterial culture using the QIAGEN DNeasy blood and tissue kit (Qiagen, Manchester, UK) following the manufacturer’s instructions. The purity of the DNA was checked using a QIAxpert spectrophotometer device (Qiagen, Manchester, UK), and the DNA concentration was determined using a Qubit™ Flex Fluorometer device (ThermoFisher Scientific, Waltham, MA, USA).

The presence of methicillin resistance was determined by detecting the *mecA* gene, which encodes for penicillin’s binding protein 2a (PBP2a). This was done using a forward primer (5′-GTAGAAATGACTGAACGTCCGATAA-3′) and a reverse primer (5′-CCAATTCCACATTGTTTCCGTCTAA-3′), which amplify a 310 bp fragment [18]. The reaction was performed in 25 µL using DreamTaq^TM^ 2X Green PCR Master Mix (Thermo Scientific, Waltham, MA, USA). The reaction setup included 1 µL of each (10 pmol) primer and 2 µL of DNA template (20 ng/µL). *S. aureus* (ATCC43300) was used as a positive control (ATCC, Manassas, Virginia, USA). The amplification process involved an initial denaturation at 95 °C for 4 min, followed by 30 cycles of 95 °C for 45 s, 56 °C for 1 min, and 72 °C for 1 min. This was followed by a final extension at 72 °C for 4 min. The final PCR product was analyzed on a 1.6% agarose gel stained with ethidium bromide and run for 60 min at 80 volts in 1X tris borate buffer (TBE) (BIOBasic, Markham, ON, Canada). The gel visualization was done using Genetools 4.3 image analysis software (Syngene, Frederick, MD, USA).

### 2.6. Whole-Genome Sequencing

Whole-genome shotgun (WGS) libraries were constructed using a QIAseq DNA FX library preparation kit (Qiagen, Manchester, UK). The input concentration was 100 ng of DNA, following the manufacturer’s protocol (Qiagen, Manchester, UK). The library was size-selected to have a 300–350 bp insert size. Sequencing was performed using the Illumina Novaseq 6000 platform with two SP flow cells (Illumina, San Diego, CA, USA). Data were quality-checked prior to analysis, with a Phred score cut-off of Q30. Bioinformatics analysis was conducted using the BactopiaV2 pipeline, focusing on *S. aureus*-specific workflows [19]. Screening for Panton–Valentine leukocidin was carried out using local blast database identification of the two gene’s, lukS and LukF, accessions (YP_002268030.1 and YP_002268029.1), respectively. For sequence type (ST) assignment, novel ST sequences were submitted to https://pubmlst.org/saureus/ (accessed on 26 September 2022), the public MLST database for *S. aureus*.

### 2.7. Statistical Analysis

The study utilized SPSS 21.0 statistical software (SPSS Inc., Chicago, IL, USA) to assess the species, sequence type, and source of isolates. The association between isolates was evaluated using the Chi-square Fisher’s exact test, with a significance level set at *p* < 0.05.

## 3. Results

**Figure 1:**
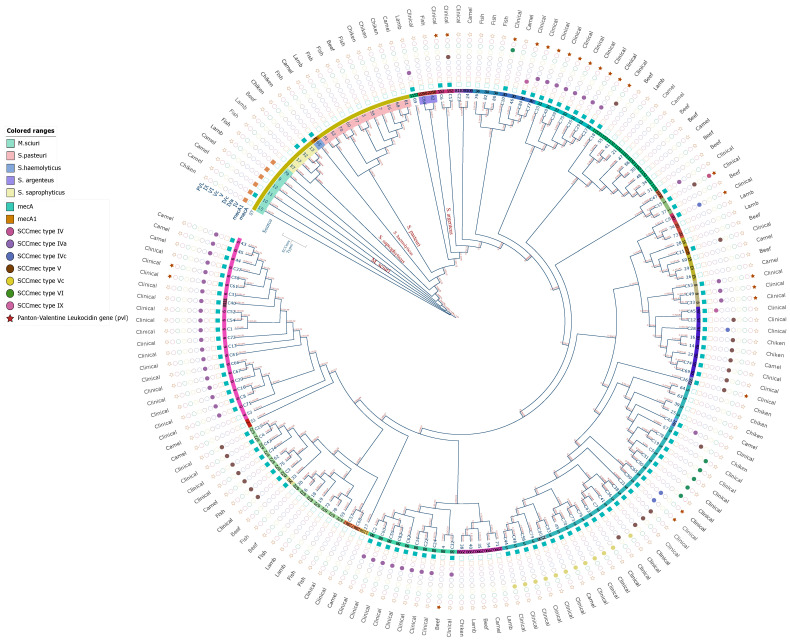
*Staphylococcus* phylogenetic tree, representing a core genome phylogeny based on single nucleotide polymorphisms (SNPs) derived from 160 *Staphylococcus* isolates, including 15 *S. aureus* clonal complexes (CC). The circles at the edge indicate the following (from inner to outer circles): 1. *S. aureus* isolate MLST sequence types indicated next to the isolate numbers; 2. The isolates numbers in the inner ranges of the circle are colored to represent different *Staphylococcus* species; 3. The colored shapes outside the circle indicate the presence of *me*cA and *mec*A1 genes, types of SCC*mec*, and the presence of the *pvl* gene; 4. *S. aureus* sources are indicated in the outer circle.

WGS was used to analyze 76 Staphylococci isolates from meat samples, including camel (23/76; 30%), fish (15/76; 20%), beef (14/76; 18%), chicken (13/76; 17%), and lamb (11/76; 15%). Out of these, 53 were *S. aureus*, with 79% (42/53) being methicillin-sensitive *S. aureus* (MSSA), and 21% (11/53) were MRSA. The remaining *Staphylococci* (23/76; 30%) belonged to other species (Figure 1). The type of meat most contaminated with MRSA was camel meat (6/11; 54%), while the least contaminated types were lamb and fish (1/11; 9%). No MRSA was detected in beef. MSSA was detected equally in camel and beef meat (12/42; 28%) and chicken was the type of meat least contaminated with MSSA (4/42; 10%). Fish meat was the most contaminated with other *Staphylococci* species (7/23; 30%), whereas beef was the least contaminated (2/23; 9%) with other *Staphylococci* species. One *S. argentous* isolate was isolated from the patients.

### 3.1. Molecular Types of Staphylococci Isolates from Meat

**Figure 2:**
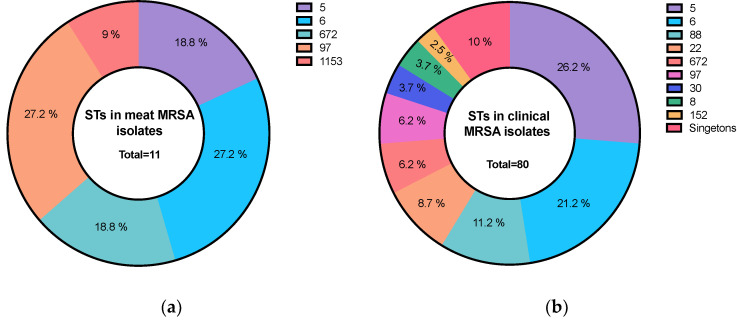
Multilocus Sequence Typing (MLST) results for *Staphylococcus aureus* in this study. (**a**) meat- and (**b**) patient-derived MRSA isolates.

The multilocus sequence typing (MLST) analysis revealed 16 diverse STs among the meat-derived *S. aureus* isolates (Table 1). Among the meat-derived MRSA isolates, 11 were assigned to five different STs. The most common clones were ST6 and ST97, found in over half (6/11; 54.5%) of the MRSA isolates. The remaining clones were ST5 (2/11; 18.18%), ST1153 (1/11; 9%), and ST672 (2/11; 18%) (Figure 2a). In contrast, meat-derived MSSA clones were more diverse, with 15 different identified STs. 

A novel sequence type, ST8109, was also identified in this study (Table 1). The predominant clones among the MSSA strains were ST1153 (9/42; 21.42%), ST672 (7/42; 16.66%), and ST2990 (5/42; 11.9%). The study also found methicillin-resistant *Staphylococci* (MRS) other than *S. aureus* among the meat isolates. All MRS isolates belonged to *Mammaliicoccus sciuri* (previously known as *Staphylococcus sciuri*) and carried the *mec*A1 gene. Methicillin-susceptible *Staphylococci* (MSS) were also detected among the meat isolates. These included *Staphylococcus pasteuri* (11/23; 47.82%), *Staphylococcus saprophyticus* (4/23; 17.39%), and one isolate each from *Staphylococcus argenteus* ST2250 and *Staphylococcus haemolyticus* ST29 (2/23; 8.69%).

### 3.2. SCCmec Types and Spa Types of S. aureus from Meat

The majority MRSA isolates carried SCC*mec* type V, observed in over half (6/11; 54.54%) of the isolates. This was followed by SCC*mec* type Iva, present in approximately a third (4/11; 36.36%) of the isolates and one isolate harboring SCC*mec* type Vc (Figure 1). The MRSA isolates exhibited seven different *S. aureus Spa* types: t12375, t903, t311, t2450, t688, t434, and t3841. These were relatively equally distributed across the MRSA isolates (Table 2). Interestingly, certain types of meat were associated with more frequent clones. For example, MRSA ST6-t2450 was more common in camel meat, while ST97-t12375 was more frequently found in chicken.

### 3.3. Molecular Types of Staphylococci Isolate from Patients

**Figure 3:**
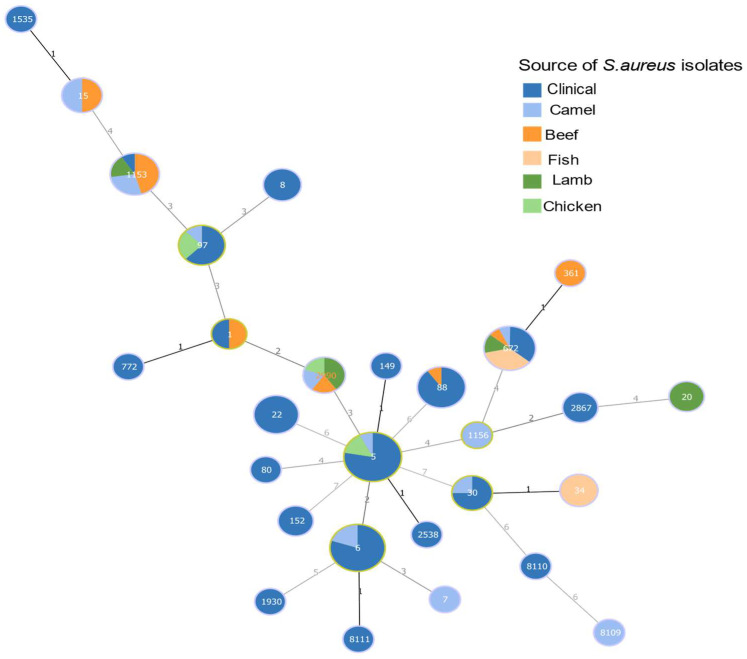
Minimum Spanning Tree (MST) analysis created using PHYLOViZ 2.0, ref. [20] representing an analysis of *S. aureus* clones based on the housekeeping genes of multilocus sequence typing (MLST) data. Each circle in the MST corresponds to a sequence type (ST). The size of each circle is proportional to the number of isolates belonging to that particular ST.

The MLST analysis identified 20 STs sequence types among the *S. aureus* isolates from patients, as shown in Figure 2b. A total of 80 MRSA isolates from patients were assigned to 18 distinct STs, including a novel sequence type, ST8111, identified in this study (Table 1). The predominant clones among the MRSA isolates were ST5 and ST6, found in nearly half of the isolates (37/80; 46%). These were followed by ST88 (9/80; 11.25%), ST22 (7/80; 8.75%), and ST97 (5/80; 6.25%), as shown in Figure 3. In contrast, there were three MSSA isolates from patients, including a novel sequence type, ST8110, also identified in this study (Table 1). Only one MSS isolate from patient was *S. argenteus* ST2250.

### 3.4. SCCmec Types and Spa Types of S. aureus from Patients

The SCC*mec* types in MRSA isolates from patients showed a diverse distribution. The most prevalent was SCC*mec* type IVa, observed in nearly half of the isolates (39/80; 48.7%). This was followed by SCC*mec* type V and its subtype Vc, present in 35% of the isolates (28/80). SCC*mec* type VI and SCC*mec* type IV with its subtype IVc were each found in 7.5% of the isolates (6/80), while SCC*mec* type IX was found in only one isolate (Figure 1). The MRSA isolates from patients exhibited 30 different *S. aureus Spa* types (Table 2). The most prevalent were *spa* types t304 and t311, accounting for 46.6% (24/30) and 43.3% (13/30) of all the *spa* types among the MRSA isolates, respectively. The remaining *spa* types were distributed among the MRSA isolates to a lesser extent (Table 2).

### 3.5. Shared Sequence Types and Spa Types among Meat and Clinical S. aureus Isolates

**Figure 4:**
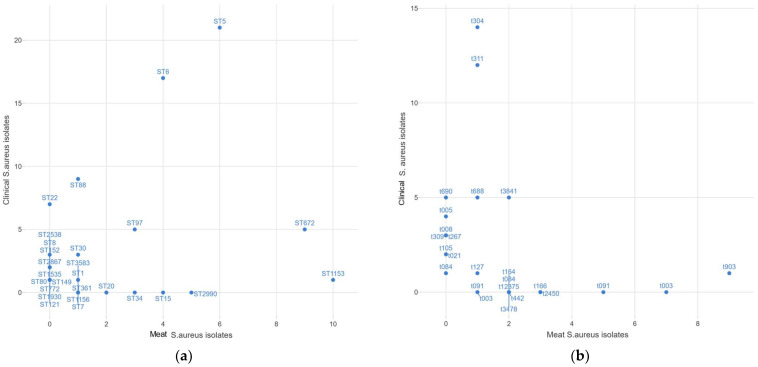
Scatter plots of *S. aureus* isolate sequence and *spa* types. (**a**) Sequence type distribution among patient- and meat-derived *S. aureus* isolates. (**b**) *spa* type distribution among patient- and meat-derived *S. aureus* isolates.

In patients, the most common *S. aureus* sequence types were ST5 (21/83; 25%) and ST6 (16/83; 19.2%), which were both also shared with meat *S. aureus* isolates ST5 (6/53; 11.3%) and ST6 (4/53; 7.5%) (Figure 4a). Concerning the *spa* types, the patient-derived *S.aureus* isolates *spa* type t304 (14/83; 16.8%) and *spa* type t311 (12/83; 14.4%) were the most common, which were also found in meat *S. aureus* isolates t304 (1/53; 1.8%) and t311 (1/53; 1.8%). The most common *spa* type was identified in meat isolate t903 (9/53; 16.9%), which was also present in clinical *S. aureus* isolates (1/83, 1.2%). Importantly, t688 and t3841 were present both in meat- and patient-derived *S. aureus* isolates (Figure 4b). The following *S. aureus* clones were present in both meat and patients: CC5/ST6-t304, CC5/ST5-t311, CC97/ST1153-t903, CC361/ST672-t3841, CC5/ST5-t688, and CC1/ST1-t127 (Table 2).

## 4. Discussion

The emergence and spread of antimicrobial-resistant *S. aureus*, particularly MRSA, poses a global challenge due to the rise in antibiotic consumption, notably in low- and middle-income countries, primarily in North Africa, the Middle East, and South Asia [21]. Additionally, excessive antibiotic usage in food-producing animals has been linked to drug-resistant bacterial infections in both animals and humans [22]. Since the global spread of LA-MRSA began in 2005, studies have primarily focused on retail meat as a possible transmission environment for MRSA [23]. Individuals who handle and prepare meat in retail settings are susceptible to MRSA colonization, indicating that retail meat is in fact a possible transmission environment for MRSA [24]. Data regarding the genetic characterization of *S. aureus*, particularly MRSA in Saudi Arabia and the genomic relationships of MRSA isolates in human, animal, and retail meat, are limited [25,26]. This study aims to address these gaps by performing a genomic analysis that includes MLST, SCC*mec*, and *spa* typing on *S. aureus* isolates from patients and meat, focusing on their occurrence, dissemination, and clonal lineages.

In this study, 76 *Staphylococci* were identified in meat, 11 of them being MRSA isolates (14.47%), indicating that retail meat might be contaminated with MRSA and could act as a reservoir for transmission. MLST analysis revealed diverse *S. aureus* clones in meat and patients, with the clonal complex (CC5) being the most dominant. Additionally, LA-MRSA clones CC97 and CC361 were also found in both patients and meat. Our data demonstrated the presence of both CA- and LA-MRSA clones in hospitals. *S. aureus* was previously reported to be present in samples of local and imported retail meat from the Riyadh region, with a prevalence rate of approximately 24% [26]. Similarly, a previous study in Riyadh city indicated that *S. aureus* was present in retail meat at a rate of 25%, and the highest contamination rate with MRSA was in camel meat (20%) [25]. Consistent with these investigations, this study found that retail meat had a 21.2% *S. aureus* contamination rate, with camel meat exhibiting the highest MRSA contamination rate (54%). In a study analyzing processed food in Riyadh, *S. aureus* prevalence was 62.6%, with MRSA prevalence at 56.3% [27]. These rates are relatively consistent across the country and are notably higher compared to other Arab countries [28].

In Saudi Arabia, the dominant MRSA clonal lineage associated with human infections has been observed to vary across different regions and over time [29]. In 2015, hospitals in Riyadh reported that the predominant MRSA clones were CC5 and CC8-ST239-III, along with other clones such as CC22-IV, ST30-IV, and CC80-IV [29]. Although the CC8-ST239-III clone was previously the most prevalent in the GCC region and Saudi hospitals, this is no longer the case [29]. Since the emergence of CA-MRSA clones, the pandemic strain CC8-ST239-III has significantly declined but is still sporadically reported in Saudi Arabia [30]. In the eastern region of Saudi Arabia, Alkharsah et al. revealed that the most identified clones from infection sites and carriers are CC80, followed by CC22 [17]. In the western region, the most prevalent MRSA lineages among clinical strains were found to be CC5, CC22, CC80, and CC30 [31]. In this study, a slight shift in MRSA common clonal linages occurred, where the most prevalent MRSA lineages in raw meat were identified as CC5, CC97, and CC361. Among the patients, the most prevalent MRSA clones were CC5, CC97, CC361, CC30, CC88, CC22, and CC8. Interestingly, none of the clinical MRSA isolates included hospital-acquired MRSA (HA-MRSA) clones since all MRSA isolates isolated from patients carried SCC*mec* types IV, IVa, IVc, V, Vc, VI, and IX. Additionally, the previously predominant clone CC8-ST239-III was not detected.

In this study, we identified MRSA clones CC5/ST5-SCC*mec*Vc-t311 and CC361/ST672-SCC*mec*V-t3841 in both patients and meat, and MRSA clones CC5/ST5-t688 and CC97ST1153-t903 in both groups with a variation in the SCC*mec* types, potentially due to several variables, including horizontal gene transfer, mutation, evolution, and different bacterial origins [32]. CC5 was the predominant MRSA clone in both groups, constituting 47.5% (38/80) of MRSA clones in patients and 45.45% (5/11) of MRSA clones in meat. The presence of SCC*mec* types IVa, V, and Vc in CC5 indicated their categorization as CA-MRSA. Notably, CC5, although prevalent in this study, was previously a less common clone, comprising less than 3% of isolated clones in a 2012 study in Riyadh [33]. The second most dominant LA-MRSA clone in meat was CC97, constituting 36% (4/11) of MRSA clones; in patients, it accounted for 7.5% (6/80). Prior to 2018, CC97 MRSA clones were less prevalent in Saudi Arabia, making up less than 1.9% in a study conducted in Dammam city and less than 9% of nosocomial infections at a tertiary care facility in Riyadh [14,15,16,17]. Another shared LA-MRSA clone detected in both patients and meat was CC361, represented by 18% (2/11) of meat MRSA isolates and 6% (5/80) of patient MRSA isolates. This clone first emerged and dominated in 2019, identified in a tertiary care facility in Riyadh, Saudi Arabia, after being reported for the first time in Kuwaiti hospitals a year prior [11,34].

This study identified two prevalent MSSA clones in meat: CC97, constituting 21.42%, and CC361, constituting 16.66% of all MSSA isolates. These clones were also found in patient MRSA isolates. In Egypt, cattle with mastitis were found to have CC1153-MSSA, while in Saudi Arabia, it was identified in humans with skin and soft tissue infections [35]. The *S. aureus*/MRSA CC1153 strain, a lesser-known Middle Eastern lineage, has been characterized in Saudi Arabia, revealing the presence of various virulent genes and indicating its potential to cause severe human and livestock infections [35]. Furthermore, MSSA clones ST361 and ST672 were identified in patients in the western region of Saudi Arabia [31]. These findings indicate that meat could serve as a source of transmission for virulent *S. aureus,* leading to infections resembling those in humans. This explains the prevalence of common MSSA lineages in both food and hospitals.

In this study, the two most prevalent MRSA clones in meat were MRSA ST6-SCC*mec*IVa-t2450 in camel meat and ST97-SCC*mec*V-t12375 in chicken. However, there is limited information on the *spa* types of MRSA clones found in meat in Saudi Arabia. In a comparative study in 2015 across two cities in Saudi Arabia and Egypt, the most frequent MRSA *spa* type in Saudi Arabia was t008, associated with CC8, which is also present in Egypt but to a lesser extent. Moreover, t084 and t085 *spa* types associated with CC15 were common in Saudi Arabia, while in Egypt, the most frequent *spa* type was t688, which is associated with CC5 [36]. Our study demonstrates a shift over time, with MRSA isolates of *spa* types t003, t903, and t091 predominating in meat, while *spa* types t304, t311, and t688 predominated among MRSA clones in patients. Patient and meat MRSA clones shared four *spa* types: t903, t311, t688, and t3841, suggesting a common evolutionary history. These *spa* types are associated with the following sequence types: ST1153-SCC*mec*IV-t903, ST5-SCC*mec*V-t311, ST5-SCC*mec*V/VI-t688, and ST672-SCC*mec*V-t3841. Additionally, the ST5-SCC*mec*V-t311 and ST5-SCC*mec*VI-t688 clones of MRSA were obtained from goats in the Eastern Province of Saudi Arabia, known to infect humans [37].

In this study, a single *S. argenteus* ST2250 isolate was found in both patient and meat samples. This species was previously categorized as *S. aureus* clonal complex 75 (CC75) [38]. It is now recognized as a clinically significant species and has been reported in numerous countries. Studies indicate that this pathogen has acquired several genes associated with livestock-associated *S. aureus* [38,39]. Additionally, six *M. sciuri* isolates carrying the *mec*A1 gene were detected in this study across all meat types except beef. *M. sciuri*, often linked with livestock, carries the *mec*A1 gene, which is a very similar gene to the *mec*A gene and confers methicillin resistance as well [40]. *S. sciuri* has been previously found in 16.66% of meat and dairy products collected in Riyadh [41].

The presence of MRSA in raw meat is well documented worldwide. For instance, in the Czech Republic, 35.4% of the raw meat samples tested positive for MRSA, while in the Netherlands, MRSA prevalence in meat was 11.9%. In the United States, MRSA prevalence in meat varied between 3% in some states and 7% in others [3,42,43,44]. The occurrence rate of MRSA in meat in this study was 14.47%, slightly higher than a previous study in 2016 that reported an MRSA rate of 13% in the various types of meat [25]. One limitation of this study is that our sample size was small and planned based on the city landscape in a limited geographical area to assess prevalence rates precisely. However, our findings are consistent with past research in the region [5,25,27]. Future research approaches should investigate the virulence genes in *S. aureus* isolates because, even though methicillin resistance is absent, virulence genes may be present and could be a source of pathogenic *S. aureus* for humans.

## 5. Conclusions

The epidemiology of *S. aureus* and its clonality have evolved due to continuous genomic changes. This study investigated the clonal flux and spread of *S. aureus* from food and its genetic relatedness to human MRSA isolates. The findings demonstrated that MRSA and MSSA clones present in meat products are shared with those found in patients. This suggests that CA- and LA-associated MRSA clones have begun to outnumber HA-MRSA clones in healthcare settings. These findings could be attributed to a lack of sanitation and veterinary monitoring, as well as the improper use of antimicrobials in the food industry. There is a clear need for further investigation to identify the distribution, genetic makeup, and antimicrobial resistance of *S. aureus* in food, as well as their impact on human health. Furthermore, regulations prescribing antimicrobial use in livestock production are recommended. This study provides a baseline for Riyadh city, which can aid future developments and comparisons for newly implemented policies. Comprehensive studies that track MRSA from livestock, food, and clinics using WGS at the local level are recommended. This would help assess the MRSA burden from a One Health prospective.

## Figures and Tables

**Table 1:** MRSA and MSSA clonal lineages and sequence types (STs) among *S. aureus* isolates from meat and patients.

Species	Clonal Complex	ST	No. (%) of Meat MRSA Isolates (n = 11)	No. (%) of Clinical MRSA Isolates (n = 80)	No. (%) of Meat MSSA Isolates (n = 42)	No. (%) of Clinical MSSA Isolates (n = 3)	No. (%) of Meat MRS Isolates (n= 6)	No. (%) of Meat MSS Isolates (n = 17)	No. (%) of Clinical MSS Isolates (n = 1)	No. (%) of Total *Staphylococci* Isolates (n = 160)
*S. aureus*	CC5	5	2 (2)	21 (23)	4 (9)	–	–	–	–	27 (17)
		6	3 (3)	16 (20)	1 (2)	–	–	–	–	20 (13)
		149	–	1 (1)	–	–	–	–	–	1 (0.6)
		2538	–	1 (1)	–	–	–	–	–	1 (0.6)
		* 8111	–	1 (1)	–	–	–	–	–	1 (0.6)
	CC1	1	–	1 (1)	1 (2)	–	–	–	–	2 (1.2)
		2990	–	–	5 (11)	–	–	–	–	5 (3)
		772	–	1 (1)	–	–	–	–	–	1 (0.6)
	CC97	97	3 (3)	5 (5)	–	–	–	–	–	8 (5)
		1153	1 (1)	1 (1)	9 (20)	–	–	–	–	11 (7)
	CC361	361	–	–	1 (2)	–	–	–	–	1(0.6)
		672	2 (2)	5 (5)	7 (14)	–	–	–	–	14 (9)
	CC30	30	–	3 (3)	1 (2)	–	–	–	–	4 (2.5)
		34	–	–	3 (7)	–	–	–	–	3 (1.8)
	CC15	15	–	–	4 (9)	–	–	–	–	4 (2.5)
		1535	–	1 (1)	–	–	–	–	–	1 (0.6)
	CC88	88	–	9 (10)	1 (2)	–	–	–	–	10 (6)
	CC22	22	–	7 (8)	–	–	–	–	–	7 (4)
	CC8	8	–	3 (3)	–	–	–	–	–	3 (1.8)
	CC80	80	–	1 (1)	–	–	–	–	–	1 (0.6)
	CC121	* 8110	–	–	–	1 (2)	–	–	–	1 (0.6)
	CC152	152	–	2 (2)	–	–	–	–	–	2 (1.2)
	CC96	1930	–	1 (1)	–	–	–	–	–	1 (0.6)
	CC7	7	–	–	1 (2)	–	–	–	–	1 (0.6)
	CC20	20	–	–	2 (5)	–	–	–	–	2 (1.2)
	ST2867	2867	–	–	–	2 (5)	–	–	–	2 (1.2)
	ST1156	1156	–	–	1 (2)	–	–	–	–	1 (0.6)
	ST8109	* 8109	–	–	1 (2)	–	–	–	–	1 (0.6)
*S. pasteuri*	ND	–	–	–	–	–	–	11 (61)	–	11 (7)
*M. sciuri*	ND	–	–	–	–	–	6 (100)	–	–	6 (4)
*S. saprophyticus*	ND	–	–	–	–	–	-	4 (22)	–	4 (2.5)
*S. argenteus*	ST2250	2250	–	–	–	–	-	1(6)	1(6)	2 (1.2)
*S. haemolyticus*	ST29	29	–	–	–	–	-	1 (6)	–	1 (0.6)

n, the number of the total isolates from the type of sample; N, the total number of *S. aureus* isolates; –, no isolates positive for this sequence type; ND, the clonal lineages for this strain not detectable; Numbers between parentheses indicate the percentage; * novel sequence types identified in this study.

**Table 2:** Meat- and patient-derived *S. aureus* isolate *spa* type frequencies and related STs.

*Spa* type	ST	Frequency	*mec*A		Isolation Source	
			MSSA	MRSA	Meat	Patients
t304	6	15 (11.4%)	1	14	1	14
t311	5	13 (9.9%)	-	13	1	12
t903	1153	10 (7.6%)	8	2	9	1
t003	* 672_* 361	8 (6.1%)	8	-	8	-
t3841	672	7 (5.3%)	-	7	2	5
t091	* 2990_* 7	6 (4.5%)	6	-	6	-
t688	5	6 (4.5%)	-	6	1	5
t690	88	5 (3.8%)	-	5	-	5
t005	22	4 (3.0%)	-	4	-	4
t2450	6	3 (2.2%)	-	3	3	-
t084	* 15_* 1535	3 (2.2%)	2	1	2	1
t166	34	3 (2.2%)	3	-	3	-
t309	22	3 (2.2%)	-	3	-	3
t008	8	3 (2.2%)	-	3	-	3
t267	97	3 (2.2%)	-	3	-	3
t12375	* 97_* 8109	2 (1.5%)	1	2	3	-
t3478	5	2 (1.5%)	2	-	2	-
t442	5	2 (1.5%)	2	-	2	-
t127	1	2 (1.5%)	1	1	1	1
t164	20	2 (1.5%)	2	-	2	-
t105	5	2 (1.5%)	-	2	-	2
t021	30	2 (1.5%)	-	2	-	2
t434	97	1 (0.7%)	-	1	1	-
t213	1156	1 (0.7%)	1	-	1	-
t605	15	1 (0.7%)	1	-	1	-
t3519	30	1 (0.7%)	1	-	1	-
t6047	15	1 (0.7%)	1	-	1	-
t1339	88	1 (0.7%)	1	-	1	-
t355	152	1 (0.7%)	-	1	-	1
t018	30	1 (0.7%)	-	1	-	1
t2297	97	1 (0.7%)	-	1	-	1
t4019	152	1 (0.7%)	-	1	-	1
t159	8110	1 (0.7%)	1	-	-	1
t345	772	1 (0.7%)	-	1	-	1
t521	97	1 (0.7%)	-	1	-	1
t2177	88	1 (0.7%)	-	1	-	1
t9736	8111	1 (0.7%)	-	1	-	1
t045	149	1 (0.7%)	-	1	-	1
t4570	1930	1 (0.7%)	-	1	-	1
t2016	2867	1 (0.7%)	1	-	-	1
t2649	88	1 (0.7%)	-	1	-	1
t1627	6	1 (0.7%)	-	1	-	1
t4494	88	1 (0.7%)	-	1	-	1
t8657	6	1 (0.7%)	-	1	-	1
t3778	2538	1 (0.7%)	-	1	-	1
t12438	88	1 (0.7%)	-	1	-	1
t319	5	1 (0.7%)	-	1	-	1

-, no isolates were positive for this *spa* type; Numbers between parentheses indicate the percentage; * The same *spa* type was carried by different *S. aureus* sequence types.

## Data Availability

The sequence data presented in this study are deposited in the European Nucleotide Archive (ENA) under accession number PRJEB64197.
